# Transcriptome analysis of Burkitt lymphoma cells treated with anti-convulsant drugs that are inhibitors of Epstein–Barr virus lytic reactivation

**DOI:** 10.1371/journal.pone.0299198

**Published:** 2024-04-18

**Authors:** Kelly L. Gorres, David M. Reineke, George Miller

**Affiliations:** 1 Department of Molecular Biophysics & Biochemistry, Yale University, New Haven, Connecticut, United States of America; 2 Department of Mathematics and Statistics and Statistics Consulting Center, University of Wisconsin-La Crosse, La Crosse, Wisconsin, United States of America; 3 Department of Pediatrics and Department of Molecular Biophysics & Biochemistry, Yale University, New Haven, Connecticut, United States of America; University of Nebraska-Lincoln, UNITED STATES

## Abstract

Herpesviruses have two distinct life cycle stages, latency and lytic replication. Epstein-Barr virus (EBV), a gamma-herpesvirus, establishes latency in vivo and in cultured cells. Cell lines harboring latent EBV can be induced into the lytic cycle by treatment with chemical inducing agents. In the Burkitt lymphoma cell line HH514-16 the viral lytic cycle is triggered by butyrate, a histone deacetylase (HDAC) inhibitor. Butyrate also alters expression of thousands of cellular genes. However, valproic acid (VPA), another HDAC inhibitor with global effects on cellular gene expression blocks EBV lytic gene expression in Burkitt lymphoma cell lines. Valpromide (VPM), an amide derivative of VPA, is not an HDAC inhibitor, but like VPA blocks induction of the EBV lytic cycle. VPA and VPM are the first examples of inhibitors of initial stages of lytic reactivation. We compared the effects of VPA and VPM, alone and in combination with butyrate, on host cellular gene expression using whole transcriptome analysis (RNA-seq). Gene expression was analyzed 6 h after addition of the compounds, a time before the first EBV lytic transcripts are detected. The results address two alternative, yet possibly complementary, mechanisms for regulation of EBV lytic reactivation. First, cellular genes that were up- or down-regulated by butyrate, but no longer altered in the presence of VPA or VPM, represent genes that correlated with EBV lytic reactivation. Second, genes regulated similarly by VPA and VPM in the absence and presence of butyrate are candidates for suppressors of EBV reactivation. Two genes upregulated by the lytic cycle inhibitors, *CHAC1* and *SLC7A11*, are related to redox status and the iron-dependent cell death pathway ferroptosis. This study generates new hypotheses for control of the latency to lytic cycle switch of EBV and provides the first description of effects of the anti-convulsant drug VPM on global human cellular gene expression.

## Introduction

**Fig 1:**
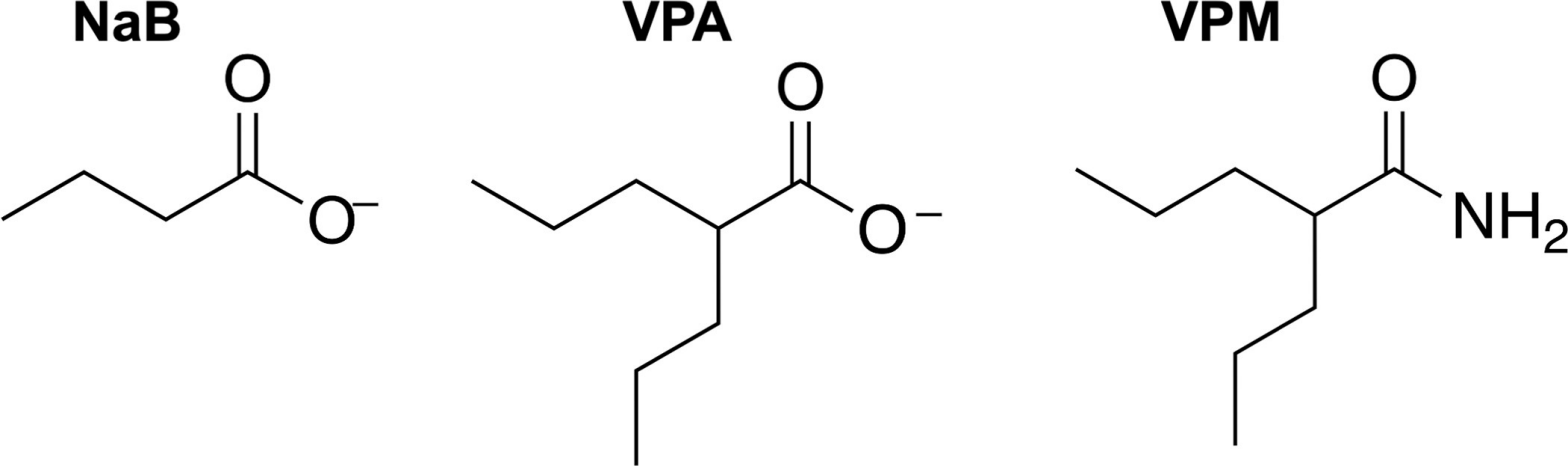
Chemical structures of small molecules: butyrate (NaB; left), valproic acid in the valproate form (VPA, middle), and valpromide (VPM, right).

Cellular gene expression is a controlled process that is responsive to various stimuli. One mechanism of regulation of gene expression is through transient post-translational modifications of histones. Chemicals and drugs that alter histone modifications alter the expression of specific and/or global gene expression. For example, sodium butyrate (NaB; Fig 1), a short-chain fatty acid, inhibits histone deacetylases leading to an increase in histone acetylation. Valproic acid (VPA; Fig 1), a medium-chain branched fatty acid, is also a histone deacetylase inhibitor (HDACi). Valpromide (VPM; Fig 1) is structurally-related to VPA, but the slight change in structure by amidation makes VPM lose its properties as an HDACi [1, 2]. While HDACi have broad effects on gene expression, all HDACi do not have exactly the same effects [3]. Interestingly, though one compound is an HDACi and the other is not, both VPA and VPM find similar applications as drugs used to treat neurological conditions, such as epilepsy and mood disorders. The anti-epileptic mechanisms of VPA and VPM are not known. Several hypotheses for their mechanisms are based on effects of the drugs on ion channels, GABA and glutamate neurotransmission, and cells signaling pathways [4, 5].

Another striking similarity between VPA and VPM is that they both inhibit reactivation of the Epstein-Barr virus (EBV) lytic cycle [6]. VPM also has antiviral activity against alpha-herpesviruses [7, 8] and cytomegalovirus, a beta-herpesvirus [9]. EBV, a double-stranded DNA gamma-herpesvirus is one of the most common human viruses, but infection usually causes either no or mild symptoms in childhood. EBV causes infectious mononucleosis when infection occurs in adolescence or adulthood. After infection EBV establishes a lifelong latent infection. EBV was discovered in Burkitt lymphoma cells and is associated with other cancers, such as Hodgkin’s lymphoma, nasopharyngeal carcinoma, and gastric carcinoma.

EBV-positive Burkitt lymphoma cells established as cell lines are useful to study the interplay of viral and host cell gene expression. Burkitt lymphoma cells infected with EBV maintain the virus in the latent state. Like all herpesviruses, EBV periodically reactivates from its latent state into its lytic life cycle. The switch is tightly regulated by controlling the expressing of two viral immediate-early genes BZLF1 and BRLF1 that encode transcription factors. The lytic cycle is reactivated in cultured cells treated with a number of different drugs, including sodium butyrate (NaB) [10] as well as agents such as phorbol esters that do not alter histone modifications. Exposure of cells to the HDACi butyrate leads to increases in the expression of thousands of cellular genes [3]. It was originally proposed that butyrate induces the expression of viral lytic genes by inhibiting HDACs. However, valproic acid (VPA), another HDAC inhibitor, does not induce the EBV lytic cycle in Burkitt lymphoma cells [3]. To investigate mechanisms by which the EBV lytic cycle is induced, the changes in cellular gene expression caused by NaB versus VPA were compared. However, since both HDAC inhibitors changed the expression of thousands of genes, it was not feasible to identify a cellular gene expression signature that was associated the capacity of VPA to inhibit initiation of EBV lytic reactivation. An approach to separate the HDACi function and teratogenicity from the anti-convulsant activity of VPA became available with the discovery of valpromide (VPM; Fig 1). VPM, the amide derivative of VPA, can be metabolized to VPA in the liver, but there are still distinct properties of VPM [11]. Although VPM is neither an HDAC inhibitor nor a teratogen like VPA, VPM is an anti-convulsant and inhibits EBV reactivation [6].

Host cellular gene expression is required for the lytic life cycle of EBV [12]. To discover cellular genes that regulate the EBV latent–lytic switch, the host cell transcriptome was assessed in cells treated with VPA or VPM in the presence and absence of butyrate, as well as butyrate alone. This experimental design investigates multiple possible mechanisms of regulation of the EBV latent-lytic switch. One mechanism identifies host genes that might have direct or downstream effects that cause EBV lytic genes to be expressed. In our data, these genes were induced by NaB alone, but not when VPA or VPM was added. Direct effects of NaB could also include the removal of a negative regulator of the lytic cycle where the host gene expression is decreased by NaB, but not in the presence of VPA or VPM. In either of these mechanisms, host genes are regulated by the inducing agent NaB, but the effects of NaB are blocked when EBV inhibitors are added. Alternatively, inhibitors of EBV lytic reactivation may up- or down-regulate cellular genes that establish a repressive environment in the cells. This mechanism may work independently of the inducing agent. Candidates for repressors of EBV reactivation were identified as the expressed genes that overlapped between cells that were inhibited in EBV reactivation (i.e VPA and VPM-treated) and cellular transcripts that were excluded from reactivated cells (butyrate-treated). These mechanisms of EBV regulation may occur simultaneously in cells. An additional medically important application of this study derives from the demonstration that VPM, a non-HDACi, elicited markedly restricted cellular gene expression by comparison to VPA. These findings should facilitate the formulation of hypotheses for unraveling the mechanisms of action of VPA and VPM in the nervous system.

## Materials and methods

### Cell culture and chemical treatments

HH514-16 human Burkitt lymphoma cells [13] were cultured in RPMI 1640 + glutamine supplemented with 8% FBS, penicillin (50 U/mL), streptomycin (50 U/mL), and amphotericin B (1 μg/mL). Cells were grown at 37°C under 5% CO_2_. Cells were subcultured to 3–4 x 10^5^ cells/mL two days prior to the experiment. The experiments were started with 1 x 10^6^ cells/mL. Sodium butyrate (NaB; Aldrich, >98%) was dissolved in water. VPA and VPM were dissolved in DMSO. Drugs were used at concentrations noted in the figures and legends. Cells were harvested 6 h and 24 h post-treatment. Cell death was measured by trypan blue staining and counting using a hemacytometer. In all experiments that investigated EBV reactivation, *>*90% of the cells were viable.

### Transcriptome sequencing

Total RNAs were isolated using the RNeasy Kit (Qiagen, Valencia, CA) according to the manufacturer’s instructions in conjunction with on-column DNase digestion to remove genomic DNA contamination. RNA from two biological replicates for each condition was analyzed. Total RNA samples were then sent to the Yale Center for Genome Analysis for whole transcriptome sequencing. Strand-specific polyA-RNA libraries were prepared by the Illumina TruSeq stranded protocol. The libraries underwent 75 bp paired-end reads using an Illumina HiSeq 2500 according to Illumina protocols, generating an average of 35 million paired-end reads per library.

### Sequencing data analysis

The sequencing data as fastq files were uploaded to the Galaxy web platform at www.usegalaxy.org [14]. T*his public server was used to process raw reads for quality control using the default parameters in FastQC and to* trim the data using the default parameters in Trimmomatic [14]. Transcript abundance counts were quantified using Salmon (v0.12.0) in Anaconda with the hg38.95 human reference transcriptome following the process in this Bioconductor vignette [15]. These data files have been deposited in the NCBI Gene Expression Omnibus (GEO) under accession number GSE135794.

Normalized and log2-transformed differentially expressed genes data from each sample were determined using DESeq2 (RRID:SCR_015687) and R packages within this Bioconductor vignette [16]. Dataset output included normalized count values, base mean, and log2FoldChange with standard error. Statistical analysis included the Wald statistic test, p-value, and adjusted p-value adjusted according to the False Discovery Rate adjustment based on the overall number of genes tested in that set. Genes with an adjusted p-value < 0.1 between the treatment and the control untreated cells [16] were used in analysis of global gene expression. The adjusted p-value was reduced to < 0.05 to reduce the false positive rate when investigating individual genes.

The similarity among the samples receiving the various treatments was assessed by calculated Euclidean distances using the sampleDists function in R and plotting the heat map with hierarchical clustering (pheatmap package in R, RRID:SCR_016418) and principal component analysis (PCA; plotPCA in R). The numeric scale in the heatmap is of Euclidean distances between samples. The color scale in the heatmap represents sample similarity. The darkest blue on the diagonal represents the Euclidean distance of 0 between a sample and itself since it is identical with itself. The darker shades mean the Euclidean distance is less, so the samples are more similar. The lighter the shade, the samples are less similar. Overlap among the differentially expressed genes in each treatment condition were listed and plotted using the VennDiagram package (RRID:SCR_002414) in R [17].

### Gene expression by RT-qPCR

Reverse transcription-quantitative polymerase chain reaction (RT-qPCR) was used to measure viral lytic and cellular gene expression. RNA was extracted from cells using the RNeasy system (Qiagen). Primers used to detect expression of EBV BZLF1 were 5’-AGCAGACATTGGTGTTCCA-3’ (forward) and 5’-CATTCCTCCAGCGATTCTG-3’ (reverse). Primers used to detect the cellular genes SLC7A11 were

5’-GGTCAGAAAGCCTGTTGT-3’ (forward) and 5’-GATGAAGATTCCTGCTCC-3’ (reverse) [18]; and CHAC1 5’-CCTTCCATCGGGGCAGCGATAAGAT-3’ (forward) and 5’-CACTTGGTATGCCACGCCCCAAGTG-3’ (reverse). The RT-qPCR utilized the iScript SYBR green RT-qPCR kit (Bio-Rad). Expression levels were normalized to 18S RNA, which is present at consistent levels among cells in all conditions. All RT-qPCR reactions were carried out on at least three biological replicates. The data are expressed as the mean ± standard deviation. Values of *P* < 0.05, calculated using R statistical analysis, were considered statistically significant.

## Results

### VPM and VPA block expression of BZLF1 in cells treated with sodium butyrate

**Fig 2:**
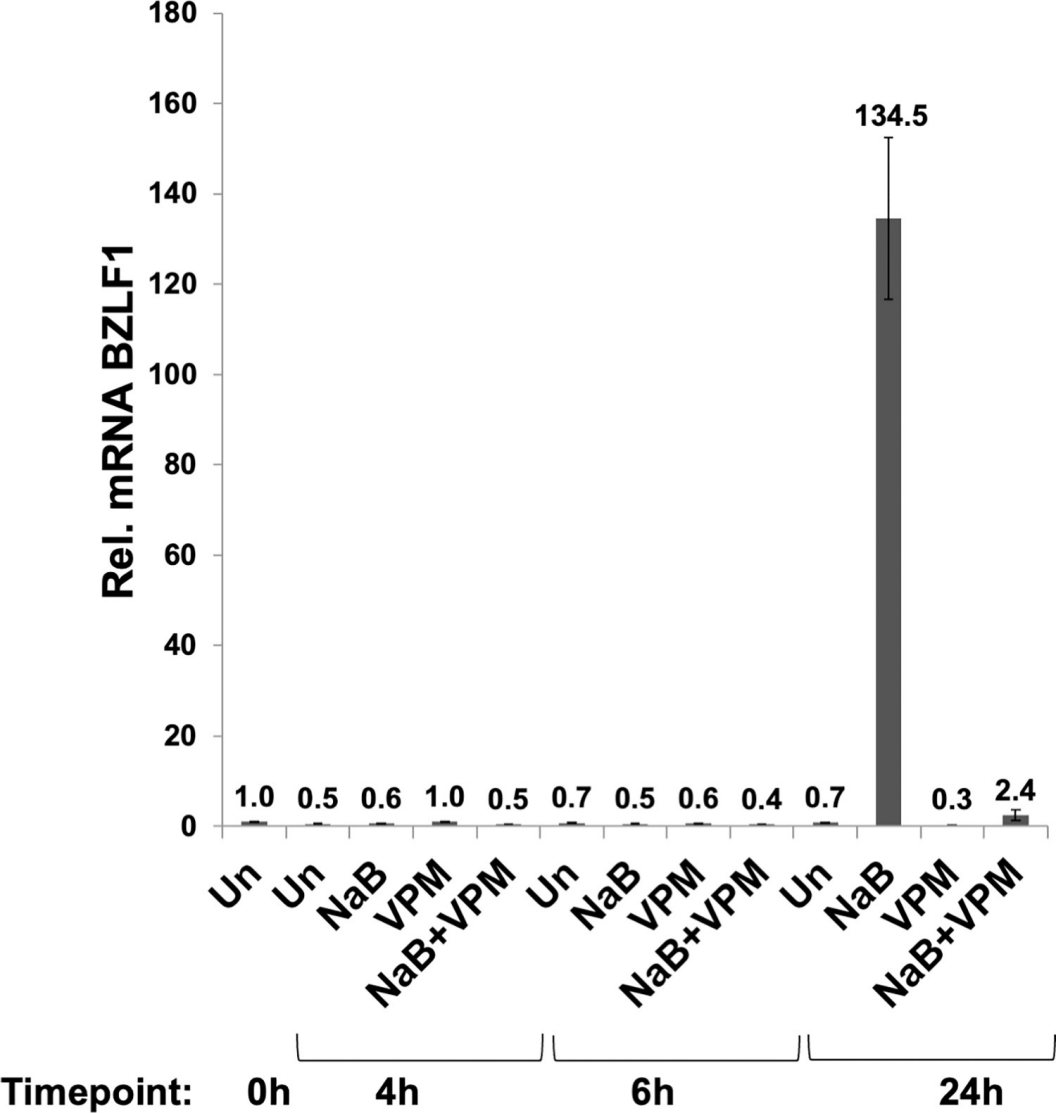
Time of expression of the EBV lytic cycle activator gene BZLF1 in HH514-16 Burkitt lymphoma cells. BZLF1 mRNA is detectable after 24 h of treatment with sodium butyrate (NaB; 3 mM), but not at 4 h or 6 h post-treatment. Valpromide (VPM; 10 mM) did not induce BZLF1 expression at any time. The induction of BZLF1 by NaB after 24 h was blocked by the addition of VPM. Data shown are the average and standard deviation of three technical replicates with three biological replicates showing the same pattern.

To confirm the effects of VPM and VPA individually, and in combination with sodium butyrate (NaB), on EBV lytic induction in the cells used for transcriptome analysis, EBV-positive HH514-16 Burkitt lymphoma cells were exposed to these two compounds for 4, 6, or 24 hours. NaB, the known inducer of the EBV lytic cycle, caused >100-fold upregulation of the EBV lytic gene BZLF1 in cells treated for 24 h, but not for 4 h or 6 h (Fig 2). This result is consistent with previous time course experiments using NaB to induce the EBV lytic cycle in HH514-16 cells where no immediate early protein expression was detected after 8 h of treatment with NaB [19]. An increase in BZLF1 and BRLF1 mRNA was observed between 6 h and 8 h [20]; ZEBRA protein was expressed after 15 hours, but not at 4, 6, or 8 hours [21]. VPM did not increase the expression of BZLF1 at any time, 4 h, 6 h, or 24 h (Fig 2). The same result was previously observed for VPA [6]. Previous data showed no lytic induction by VPM or VPA even after 48 h [6]. Thus, VPM and VPA are inhibitors of the EBV lytic cycle, as demonstrated by the lack of induction of BZLF1 in cells treated with NaB+VPM and NaB+VPA for 24 h under conditions when NaB alone strongly induces the lytic cycle (Fig 2).

### Global transcriptome analysis of cellular genes in response to VPM versus histone deacetylase inhibitors VPA and NaB

The overall goal was to characterize global gene expression patterns in cells responding to VPM, a non-HDAC inhibitor, compared to two HDAC inhibitors, VPA and NaB. However, NaB leads to induction of BZLF1 that promotes expression of downstream EBV lytic genes and causes major changes in gene expression patterns and alters metabolism in the cell [22, 23]. Therefore, identification of cellular genes that respond to VPA and VPM, as well as NaB, was conducted early after treatment, prior to the time when BZLF1 mRNA is expressed. Since the EBV transactivator gene BZLF1 was not significantly increased above the level in untreated cells at 6 h after treatment with NaB (Fig 2), changes in cellular gene expression were determined at 6 h post-treatment with NaB as an inducer and either of the two inhibitors of EBV lytic gene expression. Whole transcriptome analysis with RNA sequencing (RNA-seq) was conducted on cells treated with NaB, VPA, VPM, NaB+VPA, or NaB+VPM for 6h. The total read count was 15,250. In cells treated with the HDAC inhibitor, sodium butyrate (NaB), 605 genes were expressed significantly (adjusted p-value < 0.1) differently than untreated cells; 308 genes were upregulated (log2-fold change > 0) and 297 genes were downregulated (log2-fold change < 0) compared to untreated cells (Table 1). Similar results were found in cells treated with the HDAC inhibitor VPA. Expression of 601 genes were expressed differentially from untreated cells; of these genes 364 were upregulated and 237 were downregulated. Although there was considerable overlap between the genes regulated by NaB and VPA, there were many genes whose expression differed in cells treated with the two HDAC inhibitors. In cells treated with VPA+NaB there were 682 significantly differently expressed genes; 382 were upregulated, and 300 were downregulated compared to the untreated cells. The results from cells treated with VPM, which is not an HDAC inhibitor, were markedly different from cells treated with VPA or NaB or a combination of the two HDAC inhibitors. Only 101 genes were significantly differentially expressed; 47 genes, 0.31% of the total read count, were upregulated, and 54 genes, 0.35% of the total read count, were downregulated. The lessened effects of VPM on gene expression correlate to VPM not being an HDACi. When cells were treated with VPM in combination with NaB, the results were more similar to those with NaB alone. VPM plus NaB induced a larger number of differentially expressed genes than VPM alone: 503 genes (3.3% of genes) were affected, including 310 (2.0%) upregulated and 193 (1.3%) downregulated genes. These results show that VPM selectively regulates a smaller subset of cellular genes than does VPA. Unlike NaB and VPA, VPM does not globally suppress or activate cellular gene expression.

**Table 1:** Number of genes with significant changes in expression in 6 h drug-treated compared to untreated Burkitt lymphoma cells. A total of 15,250 genes were detected.

	Significantly^1^ differentially expressed genes	Up-regulated^2^	Down-regulated^2^
NaB	605	308	297
VPA	601	364	237
VPA+NaB	682	382	300
VPM	101	47	54
VPM+NaB	503	310	193

^**1**^Adjusted p-value <0.1

^**2**^Up-regulated = log2FoldChange >0; Down-regulated = log2FoldChange <0

**Fig 3:**
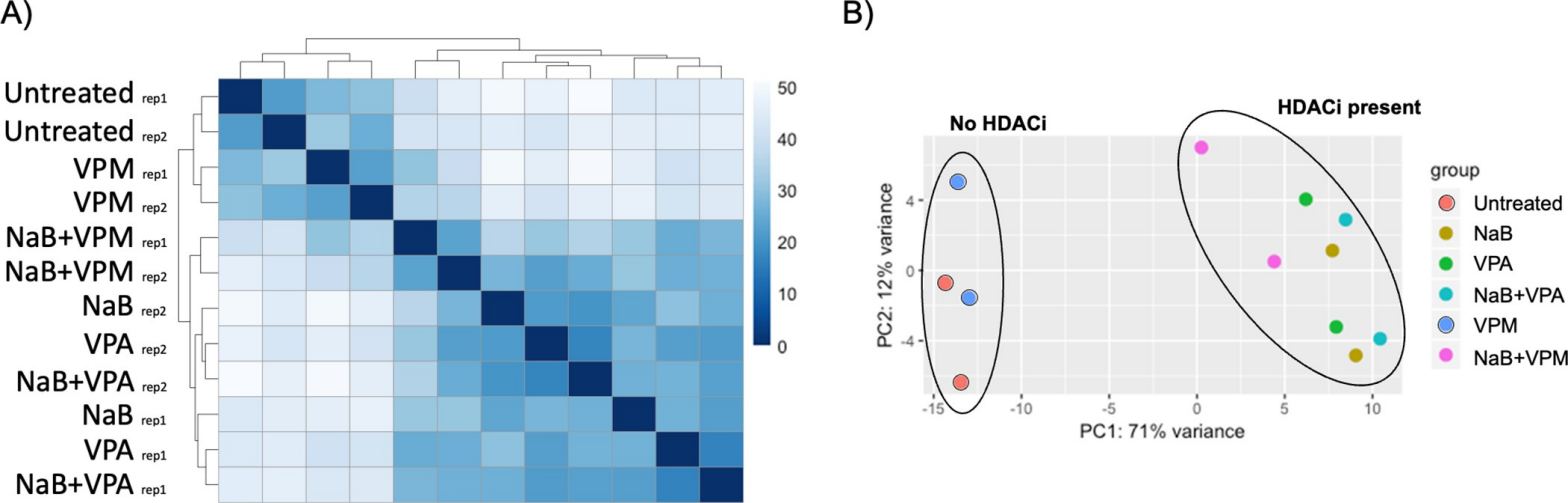
Global cellular gene expression patterns in Burkitt lymphoma HH514-16 cells. Changes in global gene expression measured by whole transcriptome analysis with RNAseq was compared pairwise in cells treated with histone deacetylase inhibitors (HDACi) sodium butyrate (NaB; 3 mM) or valproic acid (VPA; 10 mM) or the non-histone deacetylase inhibitor (non-HDACi) valpromide (VPM; 10 mM) for 6 h were compared to untreated cells. The data are illustrated in A) as a heat map with hierarchical clustering of calculated Euclidean distances with a color scale representing sample similarity, and B) via principal component analysis. Each condition had two biological replicates.

Analysis of the changes in global gene expression that resulted from treatment with the different drugs compared to the untreated cells was conducted by calculating Euclidean distances; the data are illustrated in a heat map with hierarchical clustering (Fig 3A) and principal component analysis (Fig 3B). Global gene expression in cells treated with VPM was most similar to untreated cells. Gene expression in untreated cells and VPM-treated cells clustered separately from the other conditions that included an HDAC inhibitor. The untreated and VPM-treated cells most closely resembled their own biological replicates (Fig 3A). A separate cluster formed among cells treated with any HDACi (Fig 3A and 3B). Within the group of cells exposed to any HDACi, gene expression in cells treated with NaB, VPA, or NaB+VPA were the most similar and the gene expression pattern within each group of replicates were more similar to each other than between two replicates with identical conditions. While treatment with NaB+VPM includes an HDACi, the gene expression pattern in both replicates after the addition of VPM to NaB was different than in cells treated with NaB alone. However, the addition of VPM to butyrate more closely related to conditions with HDACi than to untreated or cells treated with VPM alone. This analysis shows that global gene expression is dominated by effects of HDAC inhibition.

### Strategies for identification of cellular genes that could regulate BZLF1 expression

Regulation of biological processes, such as viral reactivation, can occur through multiple mechanisms that can function at the same time. One possibility is the up-regulation of genes that cause reactivation. Reactivation could also occur by the down-regulation of a pre-existing repressor. Opposition to these mechanisms would prevent the change of those required genes. An alternative mechanism could involve up-regulation of genes that establish a repressive environment that prevent reactivation, for example by blocking downstream effects of activators.

**Fig 4:**
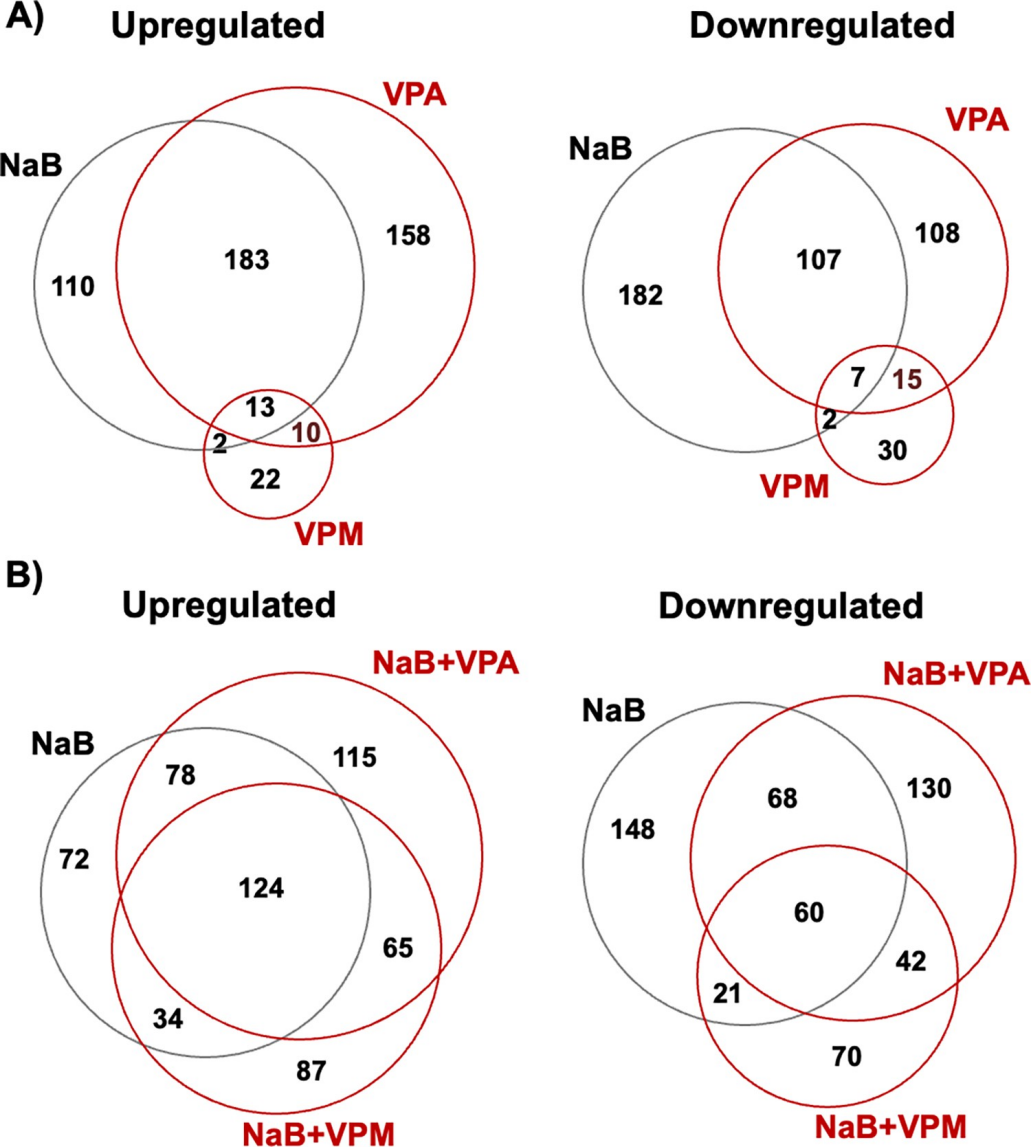
Overlap of cellular gene expression in HH514-16 Burkitt lymphoma cells treated with NaB, VPA, and VPM. The Venn diagram shows the overlap in genes significantly (padj<0.1) upregulated or downregulated from untreated cells in cells treated for 6 h with each drug. A) Comparison of cells treated with each chemical individually: butyrate (NaB; 3 mM), valproic acid (VPA; 10 mM), valpromide (VPM; 10 mM); B) Comparison of cells treated with NaB and combinations of NaB+VPA and NaB+VPM.

Gene expression altered by butyrate presumably plays a role in inducing the lytic cycle of EBV. However, since NaB is an HDACi, expression of a very large number of genes is altered by NaB (Table 2). One strategy to narrow down this pool to identify genes relevant to regulation of the EBV lytic cycle was to filter genes regulated by butyrate (padj <0.1), but not by VPA or VPM. This strategy identified 110 genes upregulated and 182 genes downregulated uniquely by butyrate (Fig 4). Further filters were applied for identification of cellular genes whose expression was changed by NaB, but no longer changed in cells treated with NaB plus VPA or NaB plus VPM, conditions under which the EBV lytic cycle is not induced. This calculation left 57 down-regulated and 20 up-regulated genes that may be involved in regulating the reactivation of the lytic cycle (S1, S2 Tables). Three of the most down-regulated and three of the most up-regulated protein-coding genes were investigated further by RT-qPCR (S1 File, S1 Fig). The three genes down-regulated by NaB were confirmed to be down-regulated by RT-qPCR, and the three genes up-regulated by NaB were also confirmed by RT-qPCR. However, down-regulation of AP5S1, TFAP4, and SDHAF2 was measured in cells treated with VPA, showing the effect is correlated to HDAC inhibition and not unique to NaB. VPM did not down-regulate AP5S1 or TFAP4, nor did it block the down-regulation caused by NaB. Similarly, VPA and NaB both induced up-regulation of ASPA3 and KLHL25, while VPM did not up-regulated these genes. The third gene up-regulated by NaB, SLC24A6, was not up-regulated by VPA alone, but VPA did not block the effect of NaB on SLC24A6 expression as it does with EBV lytic genes. In conclusion, a mechanism of viral inhibition through interfering with the changes in gene expression caused by butyrate is not identified within this set of genes. These results identify new genes that respond to NaB and VPA in HH514-16 Burkitt lymphoma cells, highlighting the prevailing effects of HDAC inhibition on cellular gene expression and the value of determining the effects of the non-HDAC inhibitor VPM on cellular gene expression by itself and in combination with butyrate.

Another strategy was to discover cellular genes that may play a role in directly repressing EBV lytic reactivation or may establish an environment that impacts the action of products of cellular genes. 25 genes were identified whose expression was altered by both inhibitors of EBV lytic reactivation, VPA and VPM, but not altered in the same direction by butyrate (Fig 4A). There were 10 genes upregulated and 15 genes downregulated by both VPA and VPM, but not by NaB. Further refinement of those gene lists was conducted by comparing genes altered by VPA or VPM alone with genes that were still altered similarly when butyrate was combined with VPA or VPM (Fig 4B). Finally, genes were identified that were up- or down-regulated in those four of the conditions in which the EBV lytic cycle is not reactivated. These conditions included genes that were commonly regulated by VPA alone, VPM alone, NaB+VPA, and NaB+VPM, but not by NaB alone which induces the EBV lytic cycle (Table 2; S2 Fig). These upregulated genes are candidates for their involvement in negatively regulating EBV lytic reactivation.

**Table 2:** Genes commonly up- or down-regulated in all conditions in which EBV lytic reactivation is blocked (VPA, NaB+VPA, VPM, NaB+VPM).

	VPA		NaB+VPA		VPM		NaB+VPM	
Up	Log2FC^1^	Padj^2^	Log2FC	Padj	Log2FC	Padj	Log2FC	Padj
CHAC1	3.03	1.6E-05	2.81	5.7E-05	2.76	1.8E-04	3.39	8.2E-07
SLC7A11	1.52	1.6E-02	1.43	2.7E-02	2.39	1.4E-05	1.72	4.9E-03
SEL1L	1.81	3.9E-06	1.73	1.1E-05	1.33	5.0E-03	1.61	1.3E-04
**Down**								
DDX20^**3**^	-2.13	7.3E-04	-2.33	1.9E-04	-1.40	1.0E-01	-2.23	6.6E-04
INPP5D^**3**^	-1.38	1.3E-04	-1.91	9.7E-09	-1.29	6.4E-04	-0.86	5.1E-02
RRM2^**3**^	-0.97	8.8E-02	-1.10	4.1E-02	-1.40	1.3E-02	-1.98	2.9E-05
HSPA8	-1.50	1.1E-06	-1.11	5.9E-04	-1.10	1.6E-03	-0.98	4.9E-03
TUBB4B^**3**^	-0.81	1.2E-03	-0.50	8.7E-02	-1.43	1.6E-10	-1.46	1.6E-10

^**1**^Log2FC: Log2 fold change relative to control untreated cells.

^**2**^Padj: Adjusted p-value

CHAC1 (ChaC glutathione specific gamma-glutamylcyclotransferase 1); SLC7A11 (solute carrier family 7 member 11); SEL1L (SEL1L adaptor subunit of ERAD E3 ubiquitin ligase); DDX20 (DEAD-box helicase 20); INPP5D (inositol polyphosphate-5-phosphatase D); RRM2 (ribonucleotide reductase regulatory subunit M2); HSPA8 (heat shock protein family A (Hsp70) member 8); TUBB4B (tubulin beta 4B class IVb)

^**3**^For these genes 3 out of the 4 treatment conditions had a Padj <0.05.

### Verification of expression of two target genes of VPM and VPA

**Fig 5:**
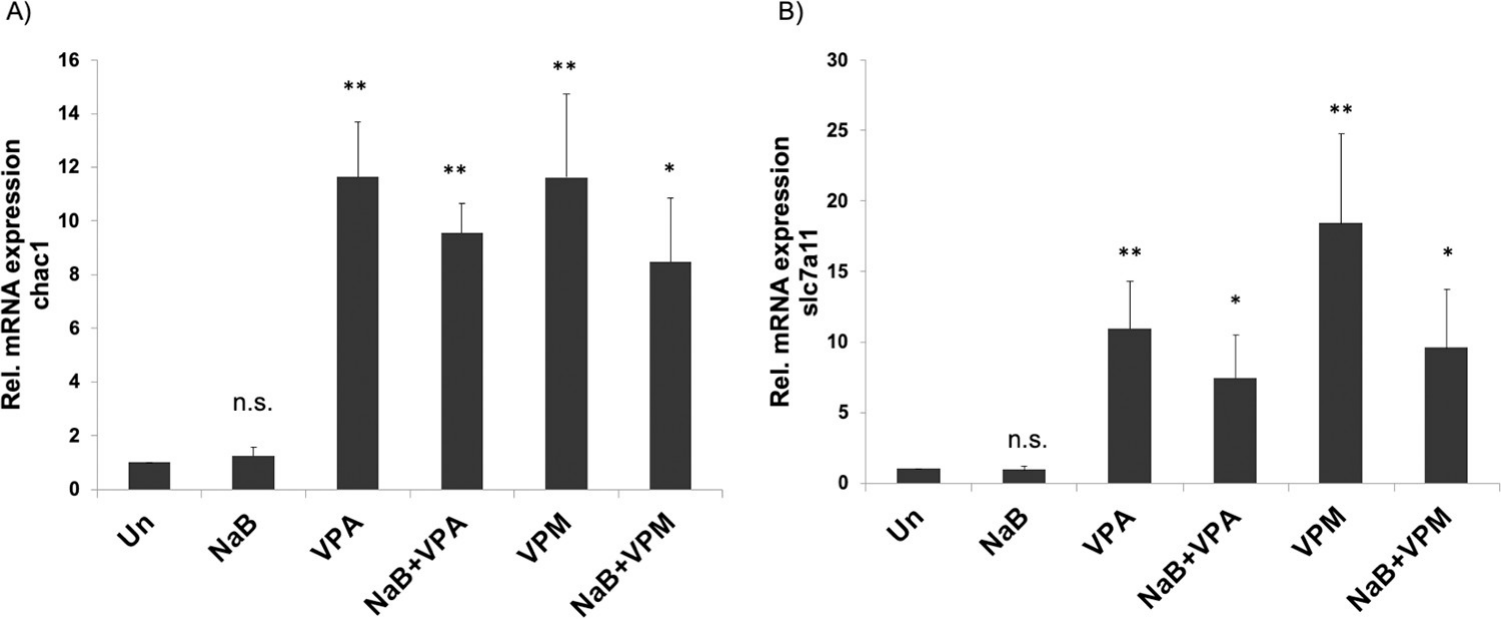
Expression of the mRNA of cellular genes CHAC1 and SLC7A11 in HH514-16 Burkitt lymphoma cells. Increased expression of *CHAC1* and *SLC7A11* is seen in cells treated for 6 h with VPA or VPM (10 mM) in the absence or presence of butyrate (NaB; 3 mM) validated by RT-qPCR. Results show the average and standard deviation of biological triplicate samples. Differences between each treatment and the untreated cells are indicated by the p-values * < 0.05, ** < 0.01, N.S. is not significantly different than the untreated control.

Two genes whose expression was increased by both VPA and VPM in the presence and absence of butyrate were *CHAC1* (Glutathione Specific Gamma-Glutamylcyclotransferase 1) and *SLC7A11* (solute carrier family 7 member 11). SLC7A11 is more functionally known as anionic amino acid transporter light chain xCT. To confirm results obtained from the high-throughput RNA sequencing experiment, additional replicates of the drug treatments of HH514-16 cells were performed and analyzed by RT-qPCR. Increased expression of *CHAC1* (Fig 5A) and *SLC7A11* (Fig 5B) was observed in all conditions that contained VPA or VPM. Expression of these two genes in cells treated with butyrate was not different than untreated cells. These genes are identified as candidates for inhibition of EBV lytic cycle reactivation.

## Discussion

### Novel findings from this study

The novel findings presented here include: 1) the general effects of small-molecule histone deacetylase inhibitors (HDACi) compared to the non-HDACi analog VPM on cellular gene expression; 2) the effects of these small molecules on EBV lytic reactivation; 3) the identification of cellular genes as possible candidates for regulators of the EBV latent–lytic switch; 4) the shared patterns of cellular gene expression induced by structurally related small molecules that have anti-convulsant and mood-altering capacities as well as anti-viral activities.

Specifically, the pattern of regulation of cellular gene expression by VPA and VPM are markedly different. VPM regulates many fewer genes than VPA, though both VPA and VPM have anti-convulsant and anti-viral properties. Nonetheless there is overlap in genes regulated by the two novel anti-viral agents VPA and VPM. A small number of genes are identified that are specifically regulated by VPA and VPM, both in the absence and in the presence of butyrate (Table 2). Among these genes are two that play a role in ferroptosis. We also identify genes whose up- or down-regulation by butyrate is blocked by VPA and VPM (S1 Table). These findings should point the way to discovery of new cellular regulators of EBV lytic reactivation and be applicable to understanding the mechanism of action of anti-epileptic agents.

### Diverse stimuli promote EBV lytic reactiveation

Viruses rely on host cell factors to complete their life cycle. Host cell protein synthesis is needed prior to EBV lytic gene expression [12]. To identify cellular genes that might be required for EBV reactivation, the response of cellular gene expression to chemical inducers of the EBV lytic cycle has been studied. The chemical stimuli that promote lytic reactivation are known to have many diverse effects on cells. Some lytic cycle activators, such as NaB and trichostatin A (TSA), are HDAC inhibitors. HDACi have widespread effects on cellular gene expression compared to non-HDACi. However, HDACi do not have identical effects since NaB and VPA are both HDACi, but regulate unique subsets of genes (Fig 4). HDAC inhibition does not appear to be a requirement for EBV reactivation since the DNA methyltransferase inhibitor azacytidine, protein kinases C agonist phorbol esters, and anti-immunoglobulins that lack HDAC inhibitory properties also induce the lytic cycle.

### Comparing the effects of VPA and VPM on cellular global gene expression

VPA and VPM are structurally very similar and both are utilized clinically as anti-convulsant drugs. The number of cellular genes whose expression is altered by VPM was much lower than the number regulated by VPA (Table 2). The data presented here show that the response of gene expression in cells treated with NaB or VPA are similar, yet still distinct, while treatment of cells with the non-HDACi VPM is more similar to untreated cells (Fig 3). This result indicates that VPM does not regulate cellular gene expression in HH514-16 cells through metabolic conversion to the HDACi VPA. Comparable findings were observed in microarray experiments that measured cellular gene expression after short treatments with the HDACi NaB or TSA, when compared to the non-HDACi, 5-aza-2’-deoxycytidine all of which induce the EBV lytic cycle switch. Cellular gene expression after treatment with 5-aza-2’-deoxycytidine clustered with the untreated control [3]. There are numerous reports of the effects of valproic acid on genome-wide cellular gene expression. In a few studies, the top hits of cellular gene expression after treatment with VPA were then tested in treatments with VPM [24, 25]. However, the global effects of VPM in human cells have not been studied. In the NCBI GEO database there is a study of embryonic and adult mouse brain gene expression using microarrays after prenatal treatment with VPM [26]. Substantial changes in cellular gene expression were caused by VPA, but not VPM, a finding similar to the results presented here (Table 2).

### Focus on a small subset of cellular genes that are regulated in common by VPA and VPM

In addition to gene products that play a role in initiating EBV reactivation, cellular gene products that inhibit the lytic cycle are also a key part of viral regulation. Genes whose expression is altered by inhibitors of EBV are candidates for repressors or establishing a repressive environment that limits the viral latent-lytic switch. This study took advantage of VPM, which is an inhibitor of EBV reactivation, but not an HDACi. Expression of many fewer genes were altered by VPM compared to VPA (Table 2). The candidate list of genes potentially involved in repression of EBV identified in this study was further narrowed by identifying genes that were similarly regulated in all conditions that contained an inhibitor, so the lytic cycle was not induced: VPA, NaB+VPA, VPM, and NaB+VPM (Table 2). This strategy greatly focused the list. Cellular genes that regulate the switch from the EBV latent to lytic cycle are potential anti-viral drug targets.

### Two candidate genes that may be involved in repressing the EBV lytic reactivation

The genes *SLC7A11* and *CHAC1* were upregulated by both inhibitors of EBV, VPA and VPM, in the presence or absence of NaB (Table 2). These changes in gene expression identified by RNA-seq were validated by RT-qPCR (Fig 5). These two genes are involved in glutathione metabolism and the redox status of the cell. It has been recently reported in other cells lines that VPA and NaB differentially alter *SLC7A11* expression. In the human placental trophoblastic choriocarcinoma BeWo cell line, VPA also increase *SLC7A11* expression [27]. NaB had the opposite effect in VPA, decreasing *SLC7A11* levels in other cell lines, such as osteosarcoma, lung cancer, colorectal adenocarcinoma, endometrial cancer [28–32]. No studies of the effects of valpromide on *SLC7A11* or any of the drugs on *CHAC1* were found in the literature.

The *SLC7A11* gene encodes the 12-pass transmembrane transporter protein (xCT) that is linked via a disulfide bridge to the single-pass transmembrane regulatory subunit *SLC3A2* (4F2hc) to form System Xc^−^, a cell-surface Na^+^-independent cystine–glutamate antiporter. The cystine-glutamate transporter brings cystine into the cell in exchange for moving a glutamate out of the cell. The imported cystine is reduced to cysteine, which is essential for production of glutathione (GSH), an anti-oxidant molecule that maintains the intracellular redox state. The increased expression of *SLC7A11* (Fig 5) may be caused by a cellular need for increased cysteine to overcome inhibition of transport activity [33, 34]. The uptake of cysteine is needed for the growth of cancer cells. Expression of the xCT subunit of the cystine-glutamate transporter, encoded by the *SLC7A11* gene, is misregulated in many tumors [35]. Since xCT is overexpressed in a number of malignancies, inhibition of xCT is one therapeutic strategy for treatment of these cancers [36].

### Role of xCT in KSHV and EBV infection

Another role for xCT has been identified as a mediator of entry of the oncogenic Kaposi’s sarcoma-associated herpesvirus (KSHV), a human gamma-herpesvirus related to EBV, into target cells [37]. Inhibition of xCT induces apoptosis in KSHV-positive primary effusion lymphoma cell lines and prevents tumor progression in a mouse model [38]. Inhibition of xCT induces KSHV lytic gene expression [38]. Entry of EBV into cells does not use xCT. A small upregulation of SLC7A11 occurred in EBV-negative 293 cells exogenously expressing the EBV lytic transactivator protein RTA [39]. Changes in SLC7A11 gene expression occur during primary EBV infection when B cells are reprogrammed and thousands of genes are regulated [40, 41]. EBV infection of B cells caused increased levels of lipid reactive oxygen species that would lead to ferroptosis, an iron-dependent programmed cell death pathway, unless mitigated by cystine transport by xCT [42]. In addition to its role in primary infection, the results herein raise the possibility that xCT plays a role in the latent-lytic switch within the EBV life cycle.

### Functions of the CHAC1 gene product

Inhibition of System Xc^−^, the cystine–glutamate transporter, leads to endoplasmic reticulum (ER) stress and the unfolded protein response, which leads to increased expression of the ER stress response gene *CHAC1* (ChaC, cation transport regulator homolog 1) [34]. *CHAC1* expression is regulated by activating transcription factor 4 (ATF4). *CHAC1* encodes the glutathione-specific gamma-glutamylcyclotransferase 1 that degrades the tripeptide glutathione into 5-oxoproline and Cys-Gly dipeptide [43]. Overexpression of *CHAC1* causes a decrease in intracellular glutathione levels [44]. Cystine starvation induces *CHAC1* expression. System Xc^−^-mediated degradation of glutathione leads to ferroptosis [45].

There are no published studies linking *CHAC1* with the Epstein-Barr virus (EBV). In response to other viral infections, *CHAC1* was induced in bat kidney cells infected with Newcastle disease virus (NDV), an enveloped, single-stranded RNA virus in the Paramyxoviridae family [46]. Paramyxoviruses typically induce a strong innate immune response. *CHAC1* expression was modestly increased by Type I interferon, but increased much more with viral infection, a finding that suggests *CHAC1* is not primarily an interferon-stimulated gene. In tests of infection with other paramyxoviruses, Nipah virus (NiV) and Hendra virus (HeV), *CHAC1* expression was not upregulated. In contrast, *CHAC1* was down-regulated in liver tissue from ducks infected with duck hepatitis A virus genotype C (DHAV-C), a member of the Picornaviridae RNA viruses [47]. *CHAC1* may be a part of pro-viral or anti-viral responses to different viruses and/or in different cell types. The role of *CHAC1* in the life cycle of EBV and other herpesviruses needs to be further explored.

No link between VPM and the *CHAC1* or *SLC7A11* genes or ferroptosis has previously been reported. The function of SLC7A11 as an antiporter releasing glutamate is intriguing in the context of studying valpromide as a drug used to treat neurological disorders since glutamate is a neurotransmitter. Further investigation of the relationship between VPM and VPA and expression of the CHAC1 and SLC7A11 genes may provide new insights into mechanisms of controlling viral latency and could expand understanding of the role of those drugs as anticonvulsants.

Identifying changes in cellular gene expression begins a novel pathway of characterization of the response of a cell to VPM. The data allow for the development of hypotheses for the mechanisms involved. These mechanisms may be tested by overexpression and knockdown experiments, though it is also possible that the process works through multiple coordinated responses rather than via the change in expression of one or a few genes. The effects may involve functions at the level of RNA and/or protein expression, protein localization and activity, and downstream changes in metabolism and cell signaling. Future work will decipher the role of cellular genes in the regulation of EBV, a possible link to epilepsy, and other effects on cell biology.

## Supporting information

S1 File Supplementary methods. (DOCX)

S1 Fig Expression of the mRNA of cellular genes altered by butyrate in HH514-16 Burkitt lymphoma cells. Cells were treated for 6 h with VPA or VPM (10 mM) in the absence or presence of butyrate (NaB; 3 mM). Levels of mRNA were measured by RT-qPCR. Results show the average and standard deviation of 5–7 biological replicates. A) AP5S1 (Adaptor Related Protein Complex 5 Sigma 1 Subunit); B) TFAP4 (Transcription factor AP-4); C) SDHAF2 (Succinate Dehydrogenase Complex Assembly Factor 2); D) ASAP3 (ArfGAP With SH3 Domain, Ankyrin Repeat And PH Domain 3); E) KLHL25 (Kelch Like Family Member 25); F) SLC24A6 (Sodium/Potassium/Calcium Exchanger 6).
(TIF)

S2 Fig Unique and overlapping expression of cellular genes after treatment with butyrate, VPA, and VPM alone and in combination. The Venn diagram shows the overlap in genes significantly (padj<0.1) upregulated or downregulated from untreated cells in HH514-16 Burkitt lymphoma cells treated for 6 h with butyrate (NaB; 3 mM), valproic acid (VPA; 10 mM), valpromide (VPM; 10 mM), NaB+VPA, and NaB+VPM.
(TIF)

S1 Table Genes up-regulated by butyrate. These 20 genes were significantly (adjusted p-value <0.05) up-regulated by butyrate compared to untreated cells as shown in the RNAseq dataset.
(XLSX)

S2 Table Genes down-regulated by butyrate. These 57 genes were significantly (adjusted p-value <0.05) down-regulated by butyrate compared to untreated cells as shown in the RNAseq dataset.
(XLSX)
