# Supplementary material for: Transcriptome analysis of Burkitt lymphoma cells treated with anti-convulsant drugs that are inhibitors of Epstein–Barr virus lytic reactivation
Source: PLoS One. 2024 Apr 18;19(4):e0299198. doi: 10.1371/journal.pone.0299198 (PMC11025866; doi:10.1371/journal.pone.0299198)
Supplement: S1 File — (DOCX) [file pone.0299198.s001.docx]

**Supplementary Methods.**

**Gene expression by RT-qPCR**

Reverse transcription-quantitative polymerase chain reaction (RT-qPCR) was used to measure cellular gene expression. RNA was extracted from cells using the RNeasy system (Qiagen). Primers used to detect the cellular genes are shown below. The most upregulated gene in the RNAseq dataset was PLLP, but the three different primers sets that were tested all failed (F) RT-qPCR quality control (QC). The primers sets for all other genes passed (P) QC. The RT-qPCR utilized the iScript cDNA synthesis kit (Bio-Rad) and GoTaq qPCR Master Mix (Promega). Expression levels were normalized to 18S RNA, which is present at consistent levels among cells in all conditions. All RT-qPCR reactions were carried out on at least three biological replicates. The data are expressed as the mean ± standard deviation.

| Gene | Forward | Reverse | QC |
| --- | --- | --- | --- |
| PLLP set 1 | GGTGATGGTCGTCGCTGTCTTC | AGAGAACGGTGGCGCTGATGTT | F |
| PLLP set 2 | GCCGAGTTCCCGTCGAAAG | ATAGGCCGGATACAGGTGGTA | F |
| PLLP set 3 | TGGGTGATGTTCGTCGCTG | GCGGTGATGTAGAGAACGGT | F |
| ASAP3 | TGTGCGGGCAATCCATAGC | GGCCAAGTTTAGGAAGCCTG | P |
| KLHL25 | TCGGTCAGTGTCCATGAGAC | GGCCTCAAAATAGCGGCTAGA | P |
| SLC24A6 | CTGACCGCTGTGACTTCATCC | GCCAGGAAACGTAGAGAGTGAC | P |
| SDHAF2 | TTCTCGACTTCGTCGCTGATG | CACCTCTGTAGAAGCGTCTGA | P |
| AP5S1 | CCCCGAATACTGAGGACACG | GACACATTGACTCTACCTGCC | P |
| TFAP4 | GTGCCCACTCAGAAGGTGC | GGCTACAGAGCCCTCCTATCA | P |
