# Supplementary figures and images for: Transcriptome analysis of Burkitt lymphoma cells treated with anti-convulsant drugs that are inhibitors of Epstein–Barr virus lytic reactivation
Source: PLoS One. 2024 Apr 18;19(4):e0299198. doi: 10.1371/journal.pone.0299198 (PMC11025866; doi:10.1371/journal.pone.0299198)

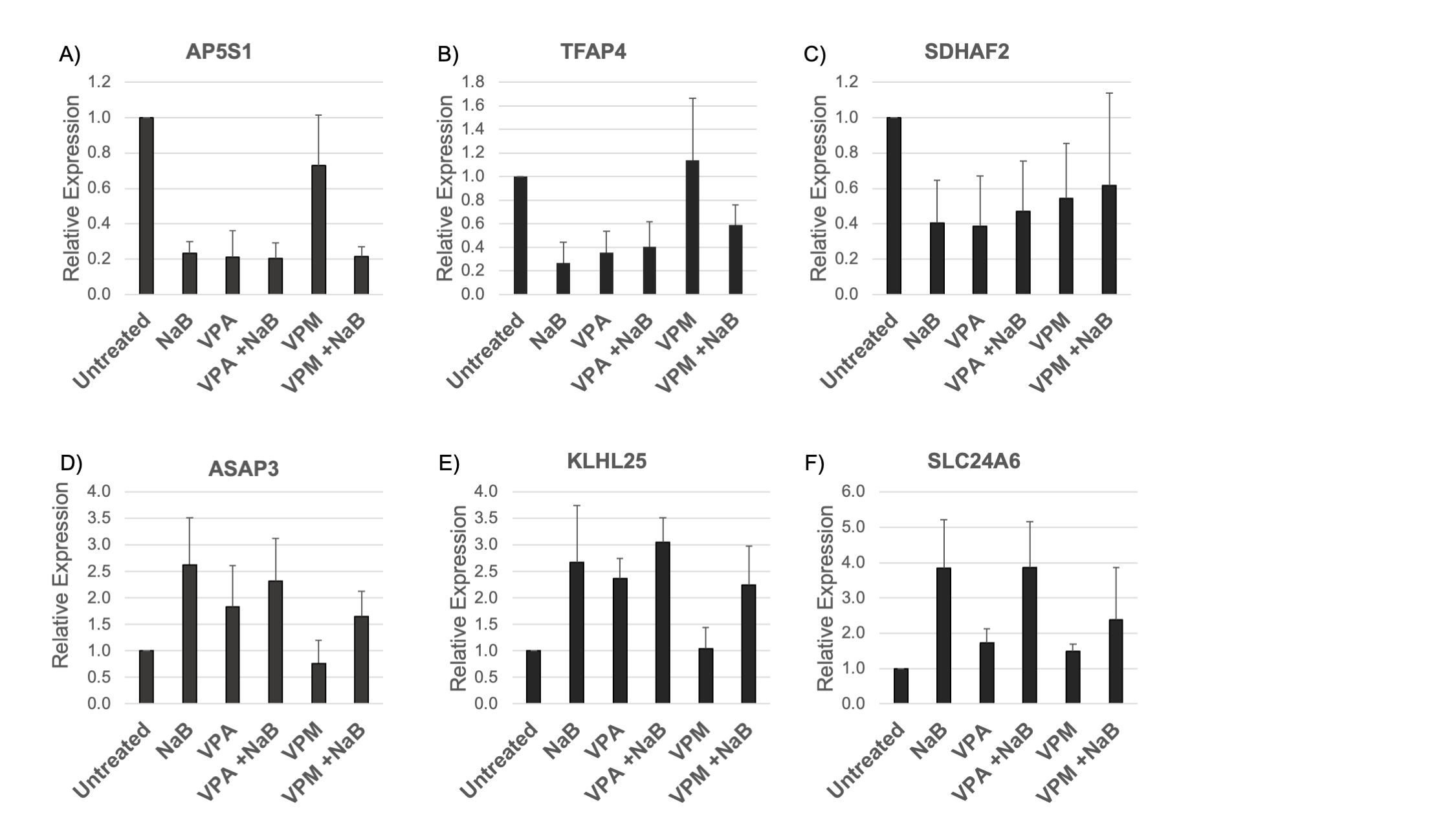

Supplement: S1 Fig — Cells were treated for 6 h with VPA or VPM (10 mM) in the absence or presence of butyrate (NaB; 3 mM). Levels of mRNA were measured by RT-qPCR. Results show the average and standard deviation of 5–7 biological replicates. A) AP5S1 (Adaptor Related Protein Complex 5 Sigma 1 Subunit); B) TFAP4 (Transcription factor AP-4); C) SDHAF2 (Succinate Dehydrogenase Complex Assembly Factor 2); D) ASAP3 (ArfGAP With SH3 Domain, Ankyrin Repeat And PH Domain 3); E) KLHL25 (Kelch Like Family Member 25); F) SLC24A6 (Sodium/Potassium/Calcium Exchanger 6). (TIF) [file pone.0299198.s002.tif]

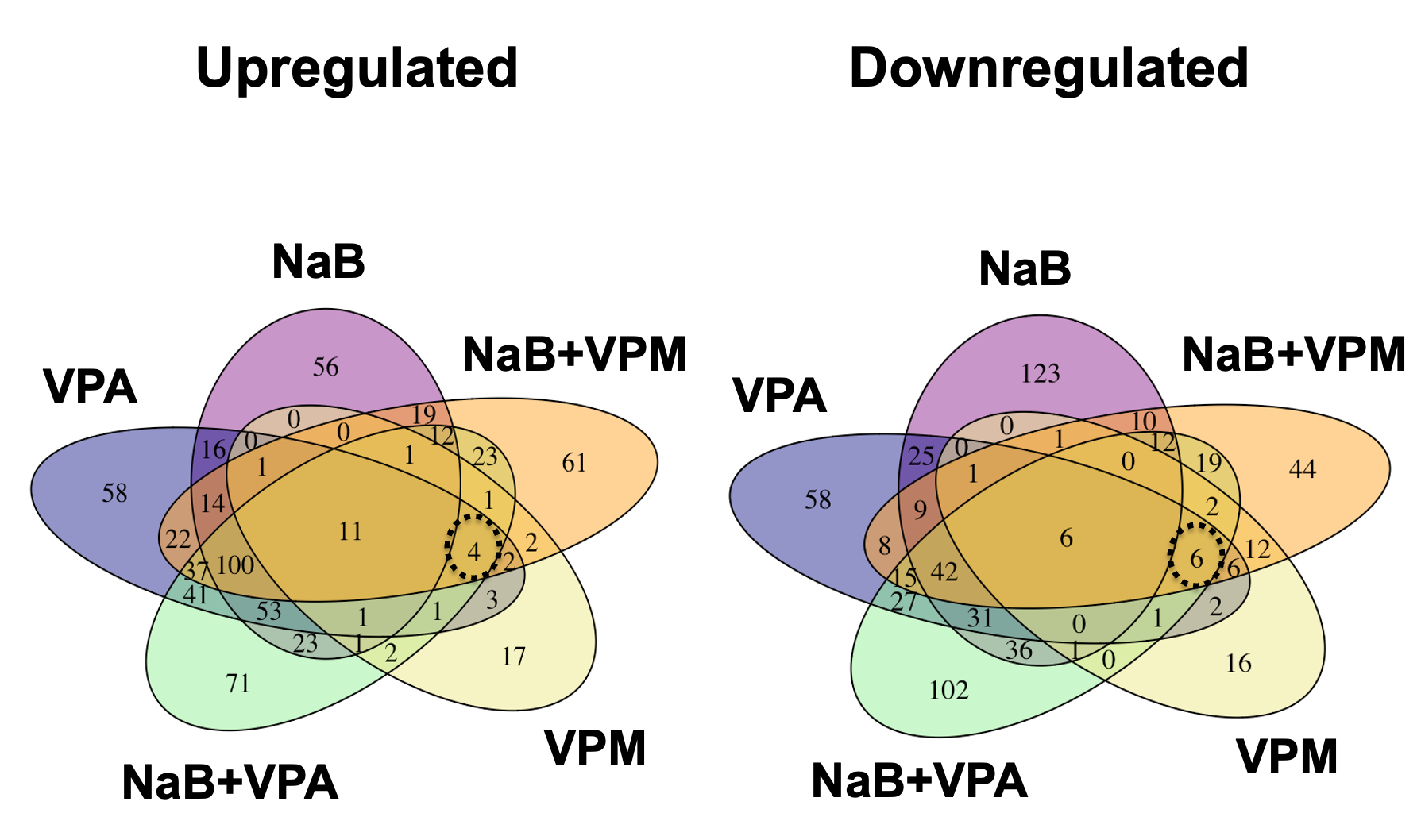

Supplement: S2 Fig — The Venn diagram shows the overlap in genes significantly (padj<0.1) upregulated or downregulated from untreated cells in HH514-16 Burkitt lymphoma cells treated for 6 h with butyrate (NaB; 3 mM), valproic acid (VPA; 10 mM), valpromide (VPM; 10 mM), NaB+VPA, and NaB+VPM. (TIF) [file pone.0299198.s003.tif]
